# COVID-19, A new challenge in the dental practice

**DOI:** 10.4317/jced.57362

**Published:** 2021-07-01

**Authors:** Francisco-Javier Silvestre, Mayte Martinez-Herrera, Cecilia-Fabiana Márquez-Arrico, Javier Silvestre-Rangil

**Affiliations:** 1 Department of Stomatology, University of Valencia, Spain; 2 Stomatology Service, Hospital Universitario Dr. Peset-FISABIO

## Abstract

**Background:**

This review was conducted in order to learn the latest information about how to prevent cross-infection of COVID-19 in dentistry. The aim of this study is offer a clinical protocol to reduce the risk of infection of COVID-19 in dental settings.

**Material and Methods:**

We carried out a review based on the PRISMA guide (Preferred Reporting Items for Systematic Reviews and Meta-Analyses). We used the following three databases: PubMed, Embase and Scopus. The search strategy was performed in the three databases applying the search terms “COVID-19 AND dental”, “COVID-19 AND dentistry”, selecting human studies published from November 2019 to May 2020. English publications regarding COVID-19 as the central topic of the research were eligible for inclusion, regardless of study design. There are very few published studies on the association between COVID-19 and dentistry, for that reason we also included the English abstract of two studies written in Chinese. The following exclusion criteria were established: animal studies and in vitro studies.

**Results:**

The search identified a total of 212 articles, of which 54 were preselected, and 23 were finally included in the review on the basis of the inclusion and exclusion criteria. We collected all the information about routes of general and oral infection, dental patient evaluation and cross-infection control in Dental Clinic in the selected studies.

**Conclusions:**

Cross infection in the dental clinic involve a very important risk due to the return to dental settings after periods of social isolation of the population after the epidemic outbreak of SARS-CoV-2. Therefore, we must take adequate and sufficient security measures to protect the patients and the dental clinic staff.

** Key words:**COVID-19, COVID-19 cross infection risk, COVID-19 prevention in Dentistry, COVID-19 in Dental Clinic.

## Introduction

Coronavirus disease (COVID-19) is an infectious disease caused by a newly discovered coronavirus. It was discovered in Wuhan, a city of China, at the end of December 2019 and up to the present time, this disease has rapidly spread in the form of a pandemic to most countries around the world (1). The World Health Organization (WHO) announced an international health emergency to this outbreak of severe pneumonia (January 30, 2020) (2). This novel coronavirus, officially named as severe acute respiratory syndrome coronavirus 2 (SARS-CoV-2), has been transmitted as a zoonosis from the reservoir in the Chinese bats (Rhinolophus sinicus) through some mammal being pangolins as the most likely intermediate host to the human (3). It is an RNA virus with a high transmission capacity between persons with a high infectivity index (R0≈2.2) (4). Due to the globalization and the ease of interconnection between countries has eased this virus, it has to spread widely around the world in a few weeks due to lack of prior immunity.

The sustained human-to-human spreads include direct transmission by droplets of saliva (5-10 μm) (cough, sneeze and droplet inhalation) and contact transmission by droplets deposition on surfaces (contact with nasal, oral and eye mucous) from the presymptomatic period to possibly after the disappearance of symptoms. Furthermore, it is suspected that there may be patients with hardly any symptoms who transmit it to a great extent (5). The virus can persist in various media from a few hours to 2-3 days (in stainless steel and plastic) (6). In addition, there may be a fecal-oral transmission and there is little data regarding a possible vertical transmission (from mothers to their newborns) (7).

To infect a cell, viruses use spike protein (SP) to bind the cell membrane, a process activated by specific cellular enzymes such as trypsin, furin, and cathepsin L. In the literature, it has been originally described that when the coronavirus enters the body, it binds to the human angiotensin-converting enzyme 2 (ACE2) receptor from the cells through the S1 subunit of SP in the membrane envelope from coronavirus (8,9). ACE2 is enzyme that is critical to regulating processes such as blood pressure, wound healing and inflammation, in a biochemical pathway called the renin-angiotensin-aldosterone system (RAAS). The ACE enzyme converts angiotensin I into angiotensin II (ANG II). The main role of ACE2 is to break down angiotensin II into other molecules that counteract the effects of ANG II.

ACE2 helps modulate the many activities of the ANG II, that increases blood pressure and inflammation, increasing damage to blood vessel linings and various types of tissue injury (8,9). In certain tissues such as the lung, a large release of inflammatory mediators (cytokine storm) can occur as an excessive and inadequate inflammatory response (10). It can cause serious tissue damage by the action of certain enzymes (proteases). This situation can lead to severe respiratory distress in the adult with very severe respiratory failure (9-11). The high affinity between ACE2 and coronavirus S1 protein suggested that the population with higher expression of ACE2 might be more susceptible to SARS-CoV-2. Current observations suggest that people of all ages are generally susceptible to this new infectious disease, but in children the infection seems to be milder (7) and in general, older age and the existence of underlying comorbidities (e.g., diabetes, hypertension, and cardiovascular disease) were associated with poorer prognosis (1). However, the pathophysiological mechanism of SARS-CoV-2 is continually being investigated and recently in early April it has been simulated that the virus “attacks” our haemoglobin A. Viral non-structural proteins (named “orf”) dissociate heme on the 1-beta chain of hemoglobin into iron and porphyrin. The attack will lead to less hemoglobin to transport oxygen to human tissues (11,12). Therefore, it is thought that the virus may first infected cells with ACE2 receptors, including immune cells, which produced antibodies. Antibodies and red blood cells will generate immune hemolysis and then the virus will attack hemoglobin, as well as it will capture porphyrin and will inhibit heme metabolism, causing systemic damage to the human body.

The disease has 3 phases. A first infection with a high viral load (during the first 7-10 days). The incubation period of COVID-19 is usually 5 days on average (2 to 14 days), which is now the commonly adopted duration for quarantine of exposed persons (13). After incubation, the majority of patients experience a viral respiratory illness with fever and dry cough, although other symptoms such as sore throat, headaches, fatigue, and other atypical symptoms, such as muscle pain, loss of smell and taste, diarrhoea and vomiting may appear (1,7). In a second phase it can give an inflammatory pulmonary chart (around the 2nd week) and it can continue for a third critical phase with a hyperinflammation and respiratory, renal failure, arrhythmias, thrombosis because the elevation of D-dimer and fibrinogen degradation products facilitates the appearance of thromboembolism, and may end in a sepsis and fatal outcome (14).

Chest tomography (CT) appears to be the most sensitive clinical diagnostic test showing bilateral pneumonia, with ground glass-like infiltrates (14). The evolution is mild in 81%, severe in 14% and critical in 5% with significant mortality. Detection should be done by PCR and antibodies against viral antigen can also be detected (15). There are rapid tests that qualitatively detect the presence of IgG and IgM antibodies against SARS-CoV-2, however the most reliable diagnostic test is the PCR method. Treatment is in line with providing early diagnosis with isolation and supportive care for patients with COVID-19. Many clinical trials are currently underway. However, some protocols have been followed so far those seem to work better clinically. Non-clinical indication drugs are used to couple them to COVID-19. Antivirals are used early in the first week as lopinavir and ritonavir. Remdesivir which is much more active has also been used. Interferon, which may have some degree of synergy effect with lopinavir and ritonavir, is associated in the treatment of severe pneumonia, but some patients have shown no improvement with interferon treatment (16). Hydroxychloroquine has been used and would have a double effect, antiviral and anti-inflammatory on patients infected with COVID-19, but its effectiveness for COVID-19 is controversial (17). On the other hand, there are anti-inflammatory drugs that have to be administered approximately one week before the patient presents the hyperinflammation chart with severe pneumonia. Corticosteroids as well as IL-6 inhibitors such as tocilizumab have been managed (16,17).

Due to the working characteristics in dental clinics, the risk of cross infection may be high among dentists and patients. For dental settings in countries that are affected with COVID-19, strict and effective infection control and action protocols are urgently needed. This article, based on relevant guidelines and recent research, introduces the essential knowledge about COVID-19 and risk of infection in dental settings and provides recommended management protocols for dental practitioners in affected areas. This review was conducted in order to answer the question: How to prevent cross-infection of COVID-19 in dentistry?

## Material and Methods

**Figure 1:**
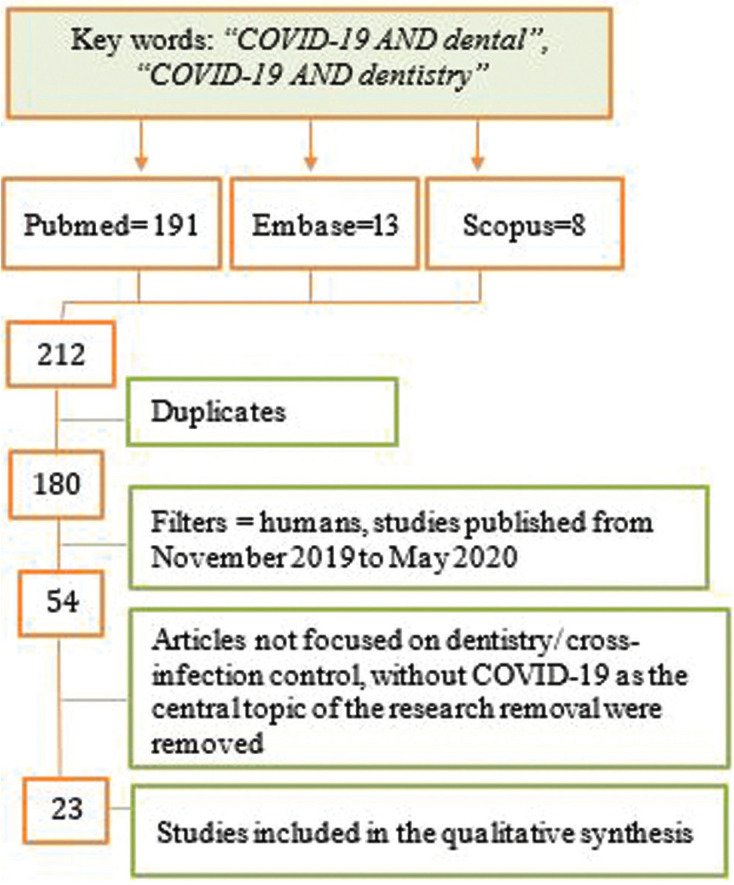
Flow chart of the selection of articles for the review.

We carried out a review based on the PRISMA guide (Preferred Reporting Items for Systematic Reviews and Meta-Analyses) (18). We used the following three databases: PubMed, Embase and Scopus. All searches and title and abstract screenings, as well as study selection, were performed independently by two investigators. Discrepancies were resolved by consensus. The search strategy was performed in the three databases applying the search terms “COVID-19 AND dental”, “COVID-19 AND dentistry”, selecting human studies published the last 6 months (from November 2019 to May 2020). Publications regarding Covid-19 as the central topic of the research were eligible for inclusion, regardless of study design. Therefore, case reports, case series, correspondences and editorials were processed in order to identify COVID-19 prevention and control of cross-infection data. English studies were included. There are very few published studies on the association between COVID-19 and dentistry, for that reason we also included the English abstract of three studies written in Chinese (12,19,20). The following exclusion criteria were established: animal studies and *in vitro* studies. Once the articles had been identified, we carried out a screening by reading abstracts. The articles whose content was not adapted to COVID-19 and its relationship with cross-infection in dentistry were eliminated. Duplicated articles were also eliminated. The flowchart corresponding to the search process is shown in Figure 1. We collected all the information about routes of general and oral infection, dental patient evaluation and cross-infection control in Dental Clinic in the selected studies.

## Results

The search identified a total of 212 articles, of which 54 were preselected, and 23 were finally included in the review on the basis of the inclusion and exclusion criteria. These 23 articles were described in  Table 1,  Table 1 cont.

**Table 1:**
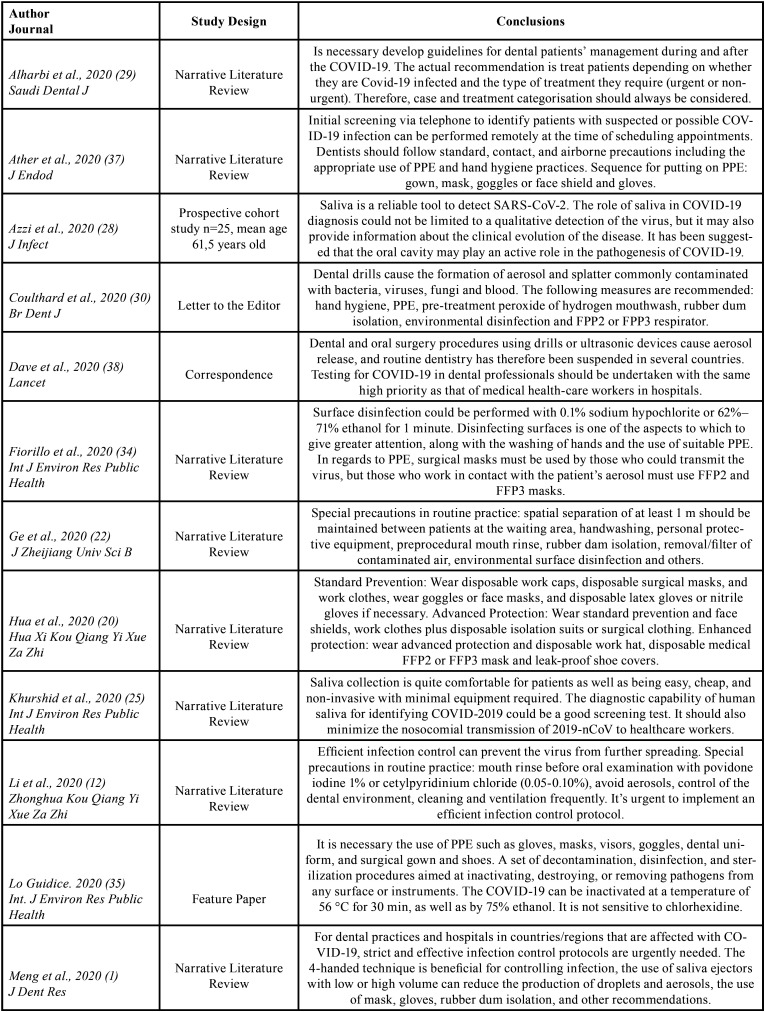
General characteristics of the studies included in the review.

**Table 1 cont:**
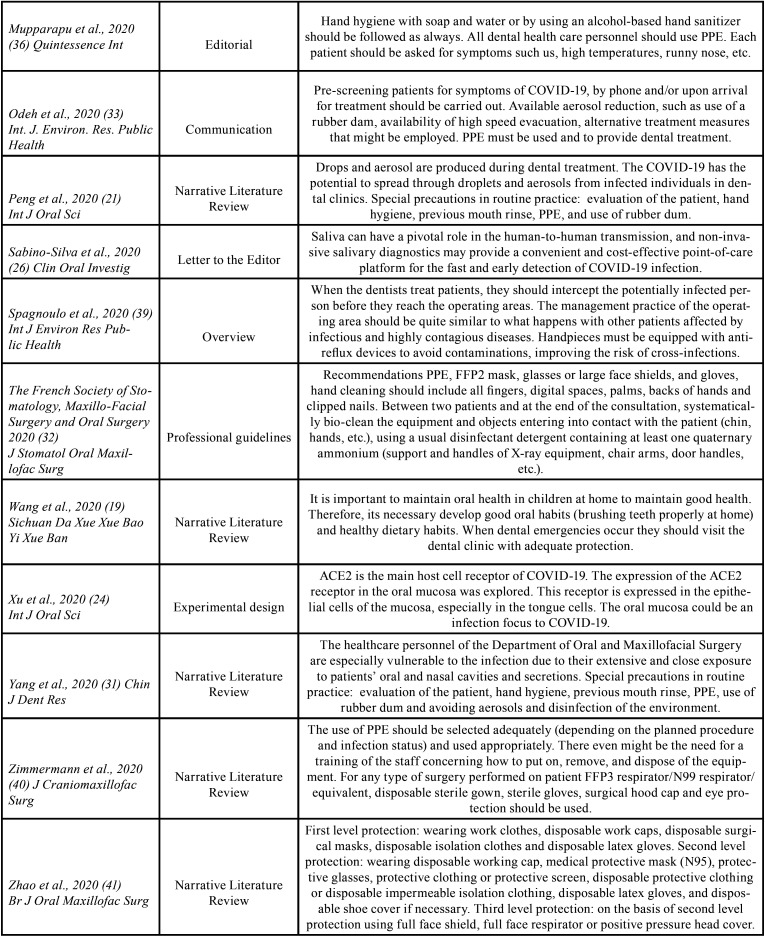
General characteristics of the studies included in the review.

-Routes of general and oral infection

The SARS-CoV-2 virus can be transmitted over short distances (<2 meters), by respiratory and saliva droplets (Plügge) and emerging evidence suggested that it may also be indirectly transmitted through contact with contaminated surfaces (8,21). In addition, although the incubation period for individuals infected has been reported to be between 5 and 14 days, it was suspected that those without an evident clinical chart could transmit the infection (5). In the dental clinic, airborne propagation has been demonstrated since many dental procedures produce aerosols when rotating instruments with water spray are used (22). The use of anti-retraction handpieces is advised and encouraged during the COVID-19 pandemic, as these anti-retraction handpieces results in a significant reduction in the backflow of bacteria and virus from oral cavity into the tubes of the handpiece in comparison with a handpiece without anti-retraction function (23).

In addition, aerosols are mixed with saliva or blood drops of the patients and bioaerosols are created. These bioaerosols are microdroplets contaminated with bacteria and viruses that can be directly projected towards the operator involving a potential risk of transmission. Likewise, these microdroplets have the potential to float in the air for a considerable amount of time or the patient can cough or sneeze and leave the work environment contaminated (22). Furthermore, there could be secondary clinical contamination through a wound from the use of sharp or cutting instruments, and even from direct contact with the mucosa.

ACE2 has been shown to be the main host receptor for virus entry. ACE2 receptors are highly expressed in the lung, colon, and kidneys among other tissues. The expression of these receptors have also been verified in the oral mucosa, particularly in the epithelial cells of the tongue, in the buccal and gingival mucosa (24). Therefore, the oral cavity could be considered a source of infectious risk for COVID-19 and should be taken into account when establishing prevention strategies for the treatment of patients in the dental clinic.

The presence of the virus in saliva has been demonstrated, so it may have a fundamental role in the oral transmission of the virus (25,26). It has been suggested that the SARS-CoV-2 can be observed in saliva by three different routes: first, the virus can get from the upper respiratory tract to the mouth. Secondly, through the crevicular fluid along with other elements from the serum. Finally, by infection of the salivary glands with direct release of the virus in the saliva through the drainage channels. In fact, salivary gland infection has been seen in animals (Macacus Rhesus) (11). Also, in experimental animal models, it has been seen specific SARS-CoV-2 A-Immunoglobulins secreted by saliva (27). It is even proposed that saliva can be a tool to detect the SARS-CoV-2 (28).

Due to the special characteristics of the dental settings, strict protective measures during dental examination and treatment of the patients must be taken to be effective to prevent the spread of infection by this novel coronavirus. Therefore, certain modifications of standard precaution and infection control regimen targeted toward COVID-19 is essential during this outbreak.

-Dental patient evaluation

Previous phone screening

When a patient requests a visit, the possibility of infection should be identified through a basic telephone evaluation by asking about symptoms. It is necessary to identify the reason for the appointment. To clarify if it is a dental emergency, we will ask to the patient: Where exactly is the pain? When did it start, was it constant/intermittent, gradual/ sudden? No patient should come without a previous screening by phone. Telephone questions should be able to identify a suspected case of COVID-19. Patients should be asked for any COVID-19 symptoms and any recent contact with positive confirmed COVID-19 individuals, or whether the patient has passed the disease and how long he/she has been discharged. Moreover, we will ask if he/she has had fever, sore throat or cough in the last 2 weeks, as well as if he/she had diarrhoea or respiratory fatigue. It is important to know the type of occupation and if it is related to healthcare or socio-healthcare. After this screening, any suspected or confirmed COVID-19 patients’ treatment should be postponed if possible for a few weeks unless it is a dental emergency (29,30).

-Clinical examination

The patient must come alone to the appointment unless he/she is a minor, a disabled person or an elderly person. He/she must come to the appointment at the agreed time to avoid having more patients in the waiting room and he/she must come with the mask on. Upon arrival at the dental clinic, he/she should rub his/her hands with a hydroalcoholic solution and then wash his/her hands with soap and water for at least 40 seconds. Dental professionals should be familiar with how this novel virus is spread, how to identify patients with COVID-19 infection, and what extra-protective measures should be adopted during the practice, in order to prevent the transmission of COVID-19 (30). In addition, every visit should be used to implement preventive measures in maintaining proper oral health at home during the outbreak, especially in children (31). Before beginning the oral examination or dental treatment, the patient should perform a mouthwash with 1% hydrogen peroxide or 0.2% povidone for 1 minute (12,32).

-Cross-infection control in Dental Clinic

Dental clinics stopped their activity except in the cases of dental emergencies in the various countries affected by the pandemic during the period of the outbreak to prevent the spread of the virus. However, after the pandemic outbreak, a restructuring will be necessary in the way of treating to patients with stricter preventive measures (32-42).

-Cross-infection control according to risk areas

The different areas of the dental clinic must be considered for proper cleaning and disinfection taking into account the possible degree of exposure to the infection. There are areas of lower exposure (low risk) such as the reception or the waiting room where the accumulation of patients should be avoided by means of correctly arranged appointments. There are other areas of greater possibility of infectious exposure (high risk), such as the dental office, the operating room and the disinfection and sterilization room (32-41). Cleaning and disinfection of all clinic surfaces is necessary, including door handles, chairs and all types of surfaces in the clinical environment. Keyboards, screens, curing lamps, ultrasound equipment, and other technical equipment within the dental cabinet should be covered with plastic wrap. It is convenient to have all the necessary equipment prepared in the work area before starting the treatment to avoid opening drawers and wardrobes when the patient is in the cabinet.

The cleaning and disinfection of the different surfaces will be carried out with a usual disinfectant for these areas (such us 70-75% ethanol solution) or with a 0.1% sodium hypochlorite solution, applying with sprays and a disposable cloth (35).

-Ventilation systems

Installation of enhanced air ventilation systems in dental cabinets can also help to facilitate the elimination of airborne pathogens and reduce the risk of infection (43). There are several methods to remove / filter contaminated air in treatment areas; one of the most commonly used devices is the high volume evacuator (HVE). It is the easiest way to remove dental aerosols as they are generated and could effectively reduce contamination caused by the operating site by 90%. The HVE is a suction device that helps remove air at a rate of up to 2.83 m3 per minute (42).

-Prevention of aerosols spread during dental treatment

If it is possible, the rubber dam should be placed because it prevents the formation of aerosols or splashes and a powerful aspiration must be placed (23). It is also convenient to use the high-speed handpiece with anti-retractive valves. A point where all current protocols coincide is the risk during dental treatment due to the formation of aerosols. These aerosols can be avoided with the use of a powerful surgical suction. In addition, studies have demonstrated that this backflow could occur during simultaneous use of other evacuation (high-volume) equipment Thus, the use of a single saliva ejector, preferably the high-volume evacuator is recommended (23,42,43).

Once the treatment is finished, the material should be left in a trough with disinfectant liquid to proceed to clean it later and once it has been thoroughly rinsed and dried, it is placed in the sealed sterilization bags following the usual procedure. The material is sterilized in the autoclave following the sterilization protocols for surgical instrument. The clinical waste generated by the treatment of patients will be considered as infectious medical waste and should go to double-layer yellow colour medical waste package bags with “gooseneck” ligation. The surface of the package bags should be marked and disposed according to the requirement for the management of infectious medical waste (21).

## Discussion

It has been studied how the dentist is a health professional who presents a high risk of exposure to the COVID-19 (32-41). Regarding the personnel clinic, the first important measure to reduce the risk of cross infection is cleaning and disinfecting hands (32-41). Hand hygiene should be carried out before putting on gloves for the clinical examination of the patient or dental procedure and after finishing the treatment. Of course, at all times the dentist or assistant will avoid touching his/her mouth, nose or eyes. Personal protective equipment (PPE) must be used appropriately, such as a mask “Filtering Face *Pi*eces class 2” (FFP2) or class 3 (FFP3), latex or vinyl long-sleeved gloves that are well suited, surgical cap, well-fitted protective glasses or goggles and face shield. All the clinic stuff should wear specific footwear with disposable wipes and a waterproof operating gown over the clinical uniform (32). All authors agree on the importance of the sequence of placement of PPE, affecting its effectiveness (32-41). The protocol for placing should be clear and all stuff must know it (32-41). PPE placement sequence: first put on shoe covers, then wash your hands for 40 seconds, then put on the waterproof gown, then the mask (FFP2 or FFP3) from behind, then the goggles, then the surgical cap, then the face shield and finally we will put on a pair of latex or vinyl long-sleeved gloves (32).

PPE removal sequence: just after the dental treatment, we will remove the face shield and then a pair of gloves to manipulate the patient’s medical history, then we will remove the gown and the surgical cap, then we will clean the other gloves with hydroalcoholic disinfectant gel and we will remove the well-fitted protective glasses and the mask, and finally we will remove the other gloves and wash our hands.

To summarise, the results of the present review supports the high risk of COVID-19 cross infection among dentists and patients. Cross infection in the dental clinic involve a very important risk due to the return to dental settings after periods of social isolation of the population after the epidemic outbreak of SARS-CoV-2 (12). Therefore, we must take adequate and sufficient security measures to protect the patients and the dental clinic staff.
